# Multimorbidity burden predicts lower likelihood of remission and higher likelihood of disease flare in patients with rheumatoid arthritis

**DOI:** 10.1136/rmdopen-2025-005577

**Published:** 2025-08-05

**Authors:** Daniel Montes, Elena Myasoedova, Chanakya Kodishala, Roslin Jose George, Andrew C Hanson, Vanessa L Kronzer, John M Davis III, Cynthia S Crowson

**Affiliations:** 1 Internal Medicine, Mayo Clinic, Rochester, Minnesota, USA; 2 Division of Rheumatology, Mayo Clinic, Rochester, Minnesota, USA; 3 Department of Quantitative Health Sciences, Mayo Clinic, Rochester, Minnesota, USA

**Keywords:** Arthritis, Rheumatoid, Outcome Assessment, Health Care, Risk Factors

## Abstract

**Objectives:**

To examine associations between multimorbidity, the social determinants of health (SDoHs) and rheumatoid arthritis (RA) flare and remission.

**Methods:**

All patients aged ≥18 years in Olmsted County, Minnesota, with incident RA in 1999–2014 were identified. Using a list of 55 chronic medical conditions, multimorbidity was defined as the presence of ≥2 conditions and substantial multimorbidity as ≥5 conditions. The Area Deprivation Index and Social Vulnerability Index (SVI) were used as proxies for adverse SDoH burden. Flare and remission were defined using Outcome Measures in Rheumatoid Arthritis Clinical Trials definitions. Mixed effects models were used to assess associations between flare/remission and multimorbidity, adverse SDoH burden and other patient characteristics.

**Results:**

This study included 659 patients with incident RA. Multimorbidity and substantial multimorbidity predicted 29% (OR:1.29, 95% CI:1.04 to 1.59) and 26% (OR:1.26, 95% CI:1.03 to 1.53) higher odds of flare, respectively. Both were associated with 34% (OR:0.66, 95% CI:0.49 to 0.90) and 33% (OR:0.67, 95% CI:0.51 to 0.90) lower odds of remission, respectively. SVI predicted 8% lower odds of remission for every 0.1 increase above 0.3 (OR:0.92, 95% CI:0.85 to 0.99). Flare was also associated with female sex, smoking, younger age and shorter disease duration, but not seropositivity. Remission was also associated with male sex, never smoking, older age and longer disease duration, but not seropositivity.

**Conclusions:**

Multimorbidity predicts higher odds of RA flare and lower odds of remission. Adverse SDoH burden predicts lower odds of remission. These findings have the potential to inform disease prognostication and clinician interventions.

**WHAT IS ALREADY KNOWN ON THIS TOPIC**

- Despite advances in treatment strategies, disease activity in rheumatoid arthritis (RA) remains difficult to control in some patients.
- Patients with RA have a higher prevalence of multimorbidity than patients without RA.

**WHAT THIS STUDY ADDS**

- This study examines multimorbidity and RA disease activity and is a first to specifically examine associations with disease flare. We noted that patients with multimorbidity had higher odds of experiencing disease flare and lower odds of achieving remission.
- Existing data on seropositivity and poor disease control are variable. Our study suggests seropositivity is not significantly associated with disease flare or remission.
- This study reinforces existing literature demonstrating the associations between female sex, smoking status, adverse social determinants of health burden and poor RA disease activity control.

**HOW THIS STUDY MIGHT AFFECT RESEARCH, PRACTICE OR POLICY**

- Our study is the first to our knowledge to examine the association between multimorbidity and disease flare in patients with RA. This highlights potential for multimorbidity to be used as a prognostic factor that can inform medical decision making for patients with RA.

## Introduction

 Multimorbidity, defined as the presence of two or more medical comorbidities, has been reported to be present in up to 86% of patients with rheumatoid arthritis (RA) and has also been shown to be more prevalent in patients with RA than patients without RA.^1^,^3^ The presence of additional medical comorbidities complicates the management of patients with RA, often necessitating greater coordination of care across multiple providers and adding uncertainty to medical decision making as the multimorbid patient is often absent in homogenous, tightly controlled clinical trials. Unsurprisingly, multimorbidity has been associated with increased mortality, poorer quality of life and increased healthcare utilisation.^4^ Drivers of multimorbidity are unclear, but considerable evidence has correlated lesser socioeconomic status and higher multimorbidity.^5^ Interestingly, like multimorbidity, adverse social determinants of health (SDoH) are also common among patients with RA.^6^

Regarding outcomes in RA, consensus guidelines endorse treat-to-target strategies, aiming for remission or low disease activity (LDA). Despite the increasing number of treatment options for RA, achieving remission or LDA remains challenging for 30–50% of patients with RA.^7 8^ Furthermore, even in patients who have achieved disease quiescence, recurrence can be experienced in the form of flares. Several predictors for high RA disease activity, disease flares and poor response to therapy have been previously described. For example, several studies have found that seropositivity is associated with a higher radiologic severity of RA compared with seronegative patients.^9 10^ There have also been reports of female sex, obesity and smoking status being associated with poorer remission rates.^11^ Existing data on RA disease activity and multimorbidity suggest a negative prognosis, with multimorbidity clusters such as cardiovascular disease, mental health disorders and substance use disorders being associated with poorer disease control.^12^ Few studies, however, have examined general multimorbidity, and those that have are limited by smaller sample sizes. Identification of multimorbidity may also be limited. Recently, our group, led by Crowson *et al*, identified 55 chronic medical conditions by extending the capture period in a population-based cohort, identifying additional comorbidities with high prevalence and adding to previously accepted lists of 40–44 comorbidities.^1 2 13^ Furthermore, most studies have focused on the achievement of remission or LDA, but none to our knowledge have examined the occurrence of flares as part of an assessment of disease activity.

Given the significant prevalence of multimorbidity, we aimed to address the aforementioned gaps, as a greater understanding of its impact on RA disease course would be invaluable, as it could inform a clinician’s approach to treatment. We aimed to assess the relationship between RA disease activity (specifically achieving remission or experiencing flare) and multimorbidity. Similarly, we also aimed to assess the relationship between adverse SDoH burden and RA disease flare and remission. We hypothesised that multimorbid patients and those with higher adverse SDoH would demonstrate poorer disease control.

## Methods

This retrospective, population-based study used the resources of the Rochester Epidemiology Project (REP). The REP allows ready access to the medical records for the local population from all healthcare providers, including institutions such as Mayo Clinic, Olmsted Medical Center and their affiliated hospitals and local nursing homes. The characteristics, strengths and generalisability of the REP have been previously described, but, briefly, the REP affords complete information on the entire medical history (including prior comorbid diagnoses) of virtually all residents residing in Olmsted county, Minnesota.^14^,^16^ The demographics, distribution of morbidity and death rates in this area are like those in the Upper Midwest in the USA.^14^

### Study population

The complete inpatient and outpatient medical records for each potential case were manually reviewed by experienced nurse abstractors using the resources of the REP. This study included all residents with incident RA from 1 January 1999 to 31 December 2014. Incident RA was defined as those who fulfilled the 1987 American College of Rheumatology classification criteria for RA.^17^

### Data collection

Data on demographics and disease activity were abstracted. All included patients had a minimum of 1-year medical history. Diagnostic codes from all healthcare providers in the county were retrieved for up to 4 years before the RA incidence date. This allowed us to identify other medical comorbidities present at RA incidence date for each patient. We employed a list of 55 previously defined chronic medical conditions by Crowson *et al* and employed their methodology of using diagnostic codes alone for identifying comorbidities.^1^ We employed the standard definition of multimorbidity (MM2+) as the presence of two or more of these comorbidities. We defined substantial multimorbidity (MM5+) as the presence of five or more of these comorbidities. This number was selected to allow for better capturing of patients with multi-system multimorbidity. For example, metabolic syndrome largely clusters four classical cardiovascular comorbidities including hyperglycaemia, hyperlipidaemia, hypertension and obesity, so a minimum of 5 morbidities would be needed to ensure the capture of more than one body system.^18^ A simple count was employed in order to maximise comparability of our study to existing multimorbidity studies, as the majority of existing multimorbidity studies use a simple count to quantify multimorbidity.^19^

Measures of social deprivation were also identified at the RA incidence date. To estimate adverse SDoH burden, we employed two area-level measurements of socioeconomic status: the Area Deprivation Index (ADI) and the Social Vulnerability Index (SVI). The ADI includes 17 variables grouped into domains of income, education, employment and housing quality. ADI is reported as national percentile rankings of neighbourhoods by decreasing socioeconomic status.^20 21^ The SVI considers 16 census variables, grouped into domains of socioeconomic status, household characteristics, racial and ethnic minority status and housing and transportation. From this, a measure of overall vulnerability is generated.^22^ For the SVI, indices <0.25 are considered low vulnerability and those >0.76 are considered high vulnerability.

Using Outcome Measures in Rheumatoid Arthritis Clinical Trials definitions, we identified flare as a worsening of disease, requiring initiation, change and/or escalation of therapy^23^ and identified remission based on findings ≤1 tender/swollen joints and normal inflammatory markers.^24^ Changes in therapy for other reasons such as side effects or intolerance were not considered as a flare. Documentation of flare in the form of statements, such as ‘flare-up’ and ‘disease exacerbation’, was also accepted. For remission, documentation of ‘remission’, ‘inactive’, ‘quiet’ or ‘quiescent disease’ was also accepted. All encounters were considered for capturing disease activity changes, including in-person visits and telehealth modalities such as phone calls or patient portal message exchanges, allowing us to capture occurrences of flare between formal clinic visits.

Use of disease-modifying antirheumatic drugs (DMARDs) for the treatment of RA was determined through medical record review. Seropositivity was determined by either a positive rheumatoid factor or anticyclic citrullinated peptide antibody.

### Statistical analysis

Descriptive statistics (means, percentages, etc) were used to summarise the data. DMARD use was summarised within the first year after RA incidence using percentages and at 15 years after RA incidence using cumulative incidence adjusted for the competing risk of death at 15 years after RA incidence. Binomial mixed models with random effects accounting for multiple visits per patient, adjusted for age, sex, RA duration, seropositivity, incidence year and smoking status, were used to assess the association between multimorbidity and flare/remission. Potential non-linear effects of continuous variables were assessed using splines. Models were fitted separately for flare and remission outcomes. Results from the base model for each outcome are from a single model, including age, sex, year of incident RA, disease duration, seropositivity and smoking status. In adjusted models, separate models including all covariates from the base model were fit for each SDoH and multimorbidity definition. Analyses were performed using R V.4.2.2 (R Foundation for Statistical Computing, Vienna, Austria).

## Results

A total of 659 patients with prevalent RA were included (table 1). Overall, the median age of these patients was 55.3 years, with 459 (69.7%) being female and 585 (88.8%) being non-Hispanic white. In terms of seropositivity, 444 (67.4%) of patients were seropositive. An ADI was measurable for 658 patients, with 116 (17.6%) patients in the least disadvantaged quartile, 498 (75.7%) patients in the middle quartiles and 44 (6.7%) patients in the most disadvantaged quartile. An SVI was measurable for 642 patients, with a median SVI of 0.21, corresponding to low community social vulnerability. In terms of multimorbidity, 483 (73.3%) patients were considered to have multimorbidity (presence of two or more of 55 previously defined chronic medical conditions) and 238 (36.1%) patients were considered to have substantial multimorbidity (presence of five or more chronic medical conditions). Patients were followed for a median of 10.3 years with a median number of 13 RA-related visits during the follow-up period. The median number of visits in which the patient was found to be experiencing RA flare was three, and the median number of visits in which the patient was found to be in remission was three. The prevalences of each of the 55 previously defined chronic medical conditions are shown in table 2.

**Table 1:** Patients with incident RA between 01 January 1999 and 31 December 2014

	(n**=**659)
Age at incidence, years, median (IQR)	55.3 (44.1–66.5)
Female sex, n (%)	459 (69.7)
Race, n (%)	
Non-Hispanic white	585 (88.8)
Asian	22 (3.3)
Black/African American	20 (3.0)
Hispanic	15 (2.3)
American Indian or Alaska Native	3 (0.5)
Native Hawaiian or Pacific Islander	3 (0.5)
Mixed	7 (1.1)
Missing	4 (0.6)
Education, n (%)	
Less than high school	226 (34.3)
High School or equivalent	357 (54.2)
College or university	76 (11.5)
Area Deprivation Index, national rank, n (%)*	
<25%	116 (17.6%)
25–74%	498 (75.7%)
75%+	44 (6.7%)
Social Vulnerability Index, median (IQR)†	0.21 (0.13, 0.56)
Smoking	
Never	345 (52.4%)
Current	105 (15.9%)
Former	209 (31.7%)
Obesity (body mass index ≥30 kg/m^2^)**,** n (%)	266 (40.4%)
Seropositivity (RF and/or CCP+), n (%)	444 (67.4%)
DMARD use	
Methotrexate within the first year after RA incidence	397 (60.2%)
Methotrexate by 15 years after RA incidence‡	477 (74.0%)
Other conventional DMARDs within the first year after RA incidence	386 (58.6%)
Other conventional DMARDs by 15 years after RA incidence‡	474 (73.6%)
Biologic or targeted synthetic DMARDs within the first year after RA incidence	76 (11.5%)
Biologic or targeted synthetic DMARDs by 15 years after RA incidence‡	183 (30.9%)
Length of follow-up for flare/remission, years, median (IQR)	10.3 (6.0, 13.6)
Total visits during follow-up, median (IQR)	13 (8, 23)
Visits in flare during follow-up, median (IQR)	3 (2, 6)
Visits in remission during follow-up, median (IQR)	3 (0, 6)
Total comorbidities, median (IQR)	3 (1, 6)
Multimorbidity (MM2+), n (%)	483 (73.3)
Substantial multimorbidity (MM5+), n (%)	238 (36.1)

* n=658;

† n=642;

‡ cumulative incidence.

CCP, citrullinated peptide; DMARD, disease-modifying anti-rheumatic drugs; MM2+, two or more comorbidities; MM5+, five or more comorbidities; RA, rheumatoid arthritis; RF, rheumatoid factor.

**Table 2:** Prevalence of 55 previously defined chronic medical conditions

Morbidity	N (%)
Alcohol abuse	11 (1.7%)
Allergic rhinitis	45 (6.8%)
Anaemia	60 (9.1%)
Anxiety	46 (7.0%)
Acquired foot deformities	11 (1.7%)
Asthma	64 (9.7%)
Benign prostatic hyperplasia *	8 (1.2%)
Bipolar disorder	5 (0.8%)
Cancer, haematologic	1 (0.2%)
Cancer, solid	28 (4.2%)
Cardiac arrhythmias	39 (5.9%)
Cerebrovascular disease	19 (2.9%)
Chronic back pain	159 (24.1%)
Chronic obstructive pulmonary disease	24 (3.6%)
Chronic skin ulcers	5 (0.8%)
Congestive heart failure	12 (1.8%)
Coronary artery disease	69 (10.5%)
Dementia	2 (0.3%)
Depression	99 (15.0%)
Diabetes mellitus	66 (10.0%)
Diverticular disease	19 (2.9%)
Drug abuse	10 (1.5%)
Erectile dysfunction*	5 (0.8%)
Fibromyalgia	36 (5.5%)
Gastro-oesophageal reflux disease	58 (8.8%)
Gout	7 (1.1%)
Headaches	61 (9.3%)
Hearing loss	26 (3.9%)
Hypercalcaemia	34 (5.2%)
Hyperlipidaemia	207 (31.4%)
Hypertension	230 (34.9%)
Hypothyroidism	106 (16.1%)
Inflammatory skin disease	31 (4.7%)
Interstitial lung disease	8 (1.2%)
Leucopaenia and white blood cell disorders	12 (1.8%)
Liver disease	19 (2.9%)
Neuropathy	69 (10.5%)
Non-inflammatory gynaecologic disorders†	91 (13.8%)
Obesity	271 (41.1%)
Osteoarthritis	160 (24.3%)
Osteoporosis	37 (5.6%)
Parkinson’s disease	2 (0.3%)
Pulmonary circulation disorders	10 (1.5%)
Peptic ulcer disease	1 (0.2%)
Peripheral vascular disease	27 (4.1%)
Post traumatic stress disorder	6 (0.9%)
Renal disease	11 (1.7%)
Restless leg syndrome	22 (3.3%)
Severe vision reduction	117 (17.8%)
Sinusitis	28 (4.2%)
Sleep disorders, including sleep apnoea	65 (9.9%)
Thrombocytopaenia and other coagulation and haemorrhagic disorders	5 (0.8%)
Urinary incontinence	15 (2.3%)
Valvular heart disease	25 (3.8%)
Vitamin D deficiency	7 (1.1%)

* men only,

† women only.

We included 648 patients with at least one visit during follow-up in the binomial mixed effects analysis to evaluate the association between patient characteristics and flare or remission visits. Table 3 details associations with flare and remission visits described as ORs. With regards to flare visits, female sex (OR 1.25, 95% CI 1.03 to 1.51), current smoker status (OR 1.49, 95% CI 1.17 to 1.90), multimorbidity (OR 1.29, 95% CI 1.04 to 1.59) and substantial multimorbidity (OR 1.26, 95% CI 1.03 to 1.53) were associated with higher odds of flare visit. Older age (OR 0.88, 95% CI 0.83 to 0.94) and longer RA disease duration (OR 0.90, 95% CI 0.89 to 0.92) were associated with lower odds of flare visit. Seropositivity, former smoker status, education, ADI and SVI were not significantly associated with flare visits.

**Table 3:** Associations of patient characteristics and multimorbidity with flare or remission visits

	Flare visit OR (95% CI)	Remission visit OR (95% CI)
Base model*		
Age**,** per 10-year increase	**0.88 (0.83 to 0.94**)	**1.10 (1.00 to 1.19**)
Female Sex	**1.25 (1.03 to 1.51**)	**0.60 (0.46 to 0.79**)
Year of Incident RA, per 5-year increase	0.98 (0.96 to 1.00)	1.01 (0.98 to 1.04)
RA Disease Duration, per 5-year increase	**0.61 (0.57 to 0.65**)	**1.60 (1.50 to 1.71**)
Seropositivity	1.13 (0.94 to 1.37)	0.95 (0.72 to 1.24)
Smoking status		
Current smoker (vs never smoker)	**1.49 (1.17 to 1.90**)	**0.48 (0.33 to 0.69**)
Former smoker (vs never smoker)	1.15 (0.94 to 1.40)	**0.74 (0.55 to 0.98**)
Adjusted models**		
Education		
Less than high school (vs high school)	0.96 (0.79 to 1.16)	0.97 (0.73 to 1.28)
College or university (vs high school)	0.91 (0.68 to 1.22)	0.97 (0.64 to 1.48)
ADI		
ADI <25% (vs 25–74%)	0.96 (0.76 to 1.21)	0.85 (0.61 to 1.19)
ADI 75%+ (vs 25–74%)	0.81 (0.56 to 1.18)	0.99 (0.57 to 1.70)
SVI, per 0.1 increase above 0.3	1.03 (0.97 to 1.08)	**0.92 (0.85 to 0.99**)
Multimorbidity (MM2+)	**1.29 (1.04 to 1.59**)	**0.66 (0.49 to 0.90**)
Substantial multimorbidity (MM5+)	**1.26 (1.03 to 1.53**)	**0.67 (0.51 to 0.90**)

Bold Figures: p < 0.05

* Models were fitted separately for flare and remission outcomes. Results from the base model for each outcome are from a single model including age, sex, year of incident RA, disease duration, seropositivity and smoking status. In adjusted models, separate models including all covariates from the base model were fit for each social determinant of health and multimorbidity definition.

ADI, area deprivation index; CI, confidence interval; OR, odds ratio; RA, rheumatoid arthritis; SVI, social vulnerability index.

With regards to remission visits, female sex (OR 0.60, 95% CI 0.46 to 0.79), current smoker status (OR 0.48, 95% CI 0.33 to 0.69), former smoker status (OR 0.74, 95% CI 0.55 to 0.98), multimorbidity (OR 0.66, 95% CI 0.49 to 0.90) and substantial multimorbidity (OR 0.67, 95% CI 0.51 to 0.90) were associated with lower odds of remission visit. Medium-to-high SVIs were also associated with lower odds of remission (OR 0.92, 95% CI 0.85 to 0.99). Older age (OR 1.10, 95% CI 1.00 to 1.19) was associated with higher odds of remission visit. Seropositivity, education and ADI were not significantly associated with remission visits.

Interactions between sex and multimorbidity and adverse SDoH burden and multimorbidity were also assessed. The odds of flare and remission appear to be independent of sex in patients with multimorbidity (MM2+) as are the odds of flare in patients with substantial multimorbidity (MM5+). However, odds of remission in patients with substantial multimorbidity depend on sex (interaction p=0.046) such that odds of remission are lower in MM5+females (OR 0.57 (0.41 to 0.79)) but not among MM5+males (OR 1.02 (0.62 to 1.67)). The odds of flare and remission appear to be independent of adverse SDoH burden in patients with multimorbidity (MM2+) as are the odds of remission in patients with substantial multimorbidity (MM5+). However, the odds of flare in patients with substantial multimorbidity depend on adverse SDoH burden (interaction p=0.010) such that odds of flare are higher in MM5+patients with SVI <0.5 (OR 1.54 (1.22 to 1.94)), but not among MM5+patients with SVI >0.5 (OR 0.92 (0.66 to 1.28)).

## Discussion

This study examined a population-based cohort of patients with RA and found several patient characteristics associated with disease control. The prevalence of comorbidities in our cohort was largely in agreement with prior analyses of multimorbidity in patients with RA. Associations with poor disease control (higher odds of flare, lower odds of remission) were found with female sex, current smoker status and multimorbidity and substantial multimorbidity. Data were mixed for former smokers (significantly lesser odds of remission but comparable odds of flare) and for adverse SDoH burden (only medium-to-high SVIs were associated with significantly lower odds of remission). With regard to better disease control, older age and longer disease duration were associated with higher odds of experiencing disease remission and lower odds of experiencing disease flare.

To our knowledge, our study is the first to examine the association between multimorbidity and disease flare and was also able to reinforce previous reports on multimorbidity’s negative association with remission. This brings to light a potential prognostic factor that can readily be assessed in the clinic. Prognostic factors are useful in guiding clinical decision making because they allow for risk stratifying and facilitating therapeutic choices. Their predictive value also allows clinicians to act pre-emptively and set preventative plans and interventions into motion before complications arise. There are several prognostic factors for RA in the scientific literature, but only a few are endorsed by consensus guidelines. For example, the European Alliance of Associations for Rheumatology (EULAR) notes the presence of high acute phase reactants, high swollen joint counts, seropositivity in high titres, the presence of early erosions or the failure of two or more conventional DMARDs as high-risk features and recommends early use of biologic DMARDs in these patients.^25^ In addition to the EULAR high-risk features, real-world surveys show that clinicians also frequently consider other prognostic factors, including the presence of other comorbidities.^11^ Since clinicians are already considering comorbidities in their decision, there is potential for multimorbidity to be easily adapted into ongoing clinical practice as a prognostic factor, and doing so may capture more at-risk patients than by considering comorbidities individually.

Interestingly, our study also found that seropositivity was not significantly associated with disease flare or remission. Seropositivity, as mentioned above, is a prognostic factor in the EULAR guidelines to prompt early initiation of biologic DMARDs. This alone may explain our findings, as clinicians may be approaching the treatment of seropositive patients more aggressively given its mention in the treatment guidelines as a high-risk feature. Multimorbidity, on the other hand, has been reported to be approached in the opposite manner, with increasing multimorbidity resulting in lower odds of initiating biologic therapies.^26^ This is despite prior studies associating multimorbidity with increased RA disease activity.^12^ However, another study assessing disease activity after initiation of first biologic DMARD showed no difference between the proportion of patients achieving remission in multimorbid versus non-multimorbid groups, with comparable numbers of seropositive patients in both groups.^27^ This suggests that the differences in association with disease activity are due more to differences in the intensity with which seropositive patients and multimorbid patients are approached and highlights the importance of recognising multimorbidity in the RA patient.

With regard to SDoHs, our study yielded mixed results. Using the ADI as a proxy, there appeared to be no association between adverse SDoH burden and disease flare or remission. However, this was in contrast to the findings when SVI was used as the proxy, with medium-to-high SVIs above an SVI of 0.3 being associated with 8% reduced odds of remission for every 0.1 increase. This association would agree with a systematic review by Dey *et al* of 30 articles, 25 of which noted lower socioeconomic status to predict more active disease.^6^ Several items warrant discussion to add some context to our mixed findings. First, both the ADI and the SVI were not designed with the intent to predict individual disease outcomes, but rather to identify under-resourced areas and target them for delivery of resources.^20 22^ Second, variables considered by these indices can be imperfect measures of deprivation. For example, one study noted that housing price items considered by ADI led to many urban areas being classified as less deprived despite other characteristics suggestive of socioeconomic deprivation.^28^ SVI, of note, does not consider housing prices alone but rather housing cost burden. This may explain the disparities between SVI and ADI in our study, as we examined a geographic area largely dominated by urban zones. Thirdly, certain SDoH domains can have more impact than others, and in other rheumatologic conditions, items such as health insurance status and race appear particularly impactful.^29^,^31^ However, ADI only tangentially considers healthcare access in the form of transportation and does not factor in race, whereas SVI has items concerning health insurance status, transportation and race, potentially capturing more impactful SDoH burden. Finally, the relatively narrow distribution of social disadvantage in our cohort should be considered. In both measures, the vast majority of our patients resided in areas of medium-to-low deprivation, with only a few residing in areas considered to have the highest levels of deprivation. This may mean that our findings are understated and that our ability to readily detect associations with adverse SDoH burden is limited. Future work examining other RA cohorts, with higher geographic and socioeconomic diversity, could shed more light on this important topic.

Our study had several strengths, including the population-based cohort of manually confirmed RA cases, data on seropositivity, the comprehensive resources of the REP that capture all medical care from all providers in the community and the long length of medical history to identify comorbidities. Limitations of our study include the use of diagnostic codes to identify comorbidities and the retrospective study design. Only diagnoses brought to medical attention and included in the medical record were included. For example, in a previous publication, our group noted that the number of patients with CT findings compatible with RA-related interstitial lung disease (RA-ILD) was considerably higher than the number of patients who carried a diagnosis of RA-ILD, suggesting that patients with mild or asymptomatic disease could be missed.^32^ However, our focus on chronic medical conditions minimises the risk of missed diagnoses. Second, the retrospective nature of this study introduces some limitations. For instance, the reliance on available and unstandardised medical records limits the rigour of applicable definitions of flare and remission as could be applied in prospective, patient-oriented studies. Indeed, in the absence of clearly delineated inflammatory markers and/or tender/swollen joint counts, subjective terms such as inactive or quiet were relied on, which could have been pre-empted in a prospective study design. We were also limited to flares that came to medical attention. Furthermore, we were also limited in our ability to quantify flare severity. Additionally, we were unable to account for DMARDs due to confounding by indication in this observational study. The presence of multimorbidity could influence the therapeutic strategy (contraindication, side effects) and therefore the occurrence of flare or remission. Similarly, SDoH may impact flares through their impact on treatment accessibility. Finally, the limited diversity of the upper Midwest population (88% white) may limit generalisability of our findings to more diverse populations.

In conclusion, multimorbidity in patients with RA was associated with higher odds of disease flare and lower odds of disease remission. Furthermore, we also provided data to suggest that adverse SDoH burden predicts lower likelihood of remission. These results add to the existing literature on RA disease activity prognostic factors and are a first to examine the association between disease flare and multimorbidity. Multimorbidity shows potential to act as a prognostic factor for RA disease activity and can be readily adapted to real world clinical settings. Increased attention to multimorbidity would help identify patients at risk of poor disease control, signalling to clinicians to consider more aggressive intervention early on. Multimorbidity and the SDoH are active topics of investigation in healthcare due to their prevalence and because they add considerable burden to the patient and the healthcare system through increased fragmentation of care and necessitating greater coordination and resources. Future research on multimorbidity and SDoH in RA could benefit from further examining their association with disease activity and if addressing them (eg, via healthcare integrative strategies or health-equity directed interventions) mitigates their association with negative outcomes.

## Data Availability

Data are available upon reasonable request.
